# Supplementary material for: Regulation of dye-decolorizing peroxidase gene expression in Pleurotus ostreatus grown on glycerol as the carbon source
Source: PeerJ. 2024 May 30;12:e17467. doi: 10.7717/peerj.17467 (PMC11144388; doi:10.7717/peerj.17467)
Supplement: Supplemental Information 1 — Occurrence of putative cis-acting regulatory elements (TATA-box, CAAT-box, carbon catabolite repressor binding elements (CreA), metal responsive element (MRE), xenobiotic responsive element (XRE), nitrogen binding site (NIT2). [file peerj-12-17467-s001.docx]

**Supplementary Table 1.** Analysis of regulatory cis-elements in promoter regions of *Pleos*-*dyp* genes. Occurrence of putative cis-acting regulatory elements (TATA-box, CAAT-box, carbon catabolite repressor binding elements (CreA), metal responsive element (MRE), xenobiotic responsive element (XRE), nitrogen binding site (NIT2)

| **Gene** | **Accession ID** | **TATA-box^1^** | **CAAT-box^2^** | **CreA^3^** | **XRE^4^** | **MRE^5^** | **NIT2^6^** |
| --- | --- | --- | --- | --- | --- | --- | --- |
| ***Pleos­DyeP1*** | 62271 | 2 | 1 | 1 | 1 | 2 | 1 |
| ***Pleos*-*DyeP2*** | 1092668 | 1 | 1 | 1 | 0 | 2 | 0 |
| ***Pleos-DyeP4*** | 1069077 | 1 | 1 | 1 | 2 | 2 | 3 |

**^1^Consensus TATA box:** 5'- TATA(A/T)A(A/T) -3'

**^2^Consensus CAAT box:** 5'- GG(T/C)CAATCT -3'

**^3^Consensus CreA:** 5'- GCTGGCAAGTT -3'

**^4^Consensus XRE:** 5'- GTATGA(G/T)AGT -3'

**^5^Consensus MRE:** 5'- GGG(A/T)A(T/A)GAC -3'

**^6^Consensus NIT2:** 5'- CG(G/C)ACCGACGG -3'

For the analysis of regulatory cis-elements in promoter regions of *Pleos*-*Dy*e*P* genes, we used 2-kb upstream from start codon of each gene. These sequences were the input for MEME (Multiple Expectation Maximization for Motif Elicitation) using default parameters. The identified motifs were annotated with SMART.
