# Supplementary material for: Regulation of dye-decolorizing peroxidase gene expression in Pleurotus ostreatus grown on glycerol as the carbon source
Source: PeerJ. 2024 May 30;12:e17467. doi: 10.7717/peerj.17467 (PMC11144388; doi:10.7717/peerj.17467)
Supplement: Supplemental Information 2 — Linear growth rates (mm/d) of P. ostreatus grown on agar plates with glucose or glycerol as carbon source and supplemented with different chemical dyes. [file peerj-12-17467-s002.docx]

**Supplementary Table 2**. Mycelial growth rate. Linear growth rates (mm/d) of *P. ostreatus* grown on agar plates with glucose or glycerol as carbon source and supplemented with different chemical dyes.

| **Carbon Source** | **Dye** | **Mycelial Growth Rate (mm/day)** | **R^2^** |
| --- | --- | --- | --- |
| Glucose | Control | 2.65 | 0.95 |
|  | AYG | 5.04 | 0.93 |
|  | RBBR | 4.85 | 0.88 |
|  | AB129 | 4.75 | 0.93 |
| Glycerol | Control | 2.53 | 0.93 |
|  | AYG | 3.06 | 0.95 |
|  | RBBR | 2.58 | 0.89 |
|  | AB129 | 1.90 | 0.97 |

Kruskal-Wallis chi-squared = 4.083, *p* = 0.0433).
